# Energy Saving and Energy Generation Smart Window with Active Control and Antifreezing Functions

**DOI:** 10.1002/advs.202105184

**Published:** 2022-01-11

**Authors:** Yingchun Niu, Yang Zhou, Daxue Du, Xiangcheng Ouyang, Ziji Yang, Wenjie Lan, Fan Fan, Sisi Zhao, Yinping Liu, Siyuan Chen, Jiapeng Li, Quan Xu

**Affiliations:** ^1^ State Key Laboratory of Heavy Oil Processing China University of Petroleum Beijing 102249 China; ^2^ School of Environment and Chemical Engineering Yanshan University Qinhuangdao 066004 China

**Keywords:** active control, anti‐freezing, energy storage, energy‐saving, smart window

## Abstract

Windows are the least energy efficient part of the buildings, as building accounts for 40% of global energy consumption. Traditional smart windows can only regulate solar transmission, while all the solar energy on the window is wasted. Here, for the first time, the authors demonstrate an energy saving and energy generation integrated smart window (ESEG smart window) in a simple way by combining louver structure solar cell, thermotropic hydrogel, and indium tin oxides (ITO) glass. The ESEG smart window can achieve excellent optical properties with ≈90% luminous transmission and ≈54% solar modulation, which endows excellent energy saving performance. The outstanding photoelectric conversion efficiency (18.24%) of silicon solar cells with louver structure gives the smart window excellent energy generation ability, which is more than 100% higher than previously reported energy generation smart window. In addition, the solar cell can provide electricity to for ITO glass to turn the transmittance of hydrogel actively, as well as the effect of antifreezing. This work offers an insight into the design and preparation together with a disruptive strategy of easy fabrication, good uniformity, and scalability, which opens a new avenue to realize energy storage, energy saving, active control, and antifreezing integration in one device.

## Graphical abstract

The authors develop a revolutionary smart window with a multi‐layer louver structure, containing a silicon solar cell, thermotropic hydrogel, and ITO active layer, which combine both an energy saving and energy generation ability (ESEG smart window) with leverages high solar energy modulation together with high photoelectric conversion efficiency (PCE).

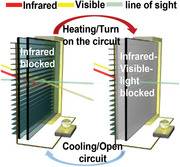

## 1 Introduction

Building accounts for 40% of total global energy consumption, while heating, ventilation, and air‐conditioning (HVAC) consume half of the building energy consumption,^[^ ^1^, ^2^, ^3^ ^]^ improving energy efficiency is critical to address this issue.^[^ ^3^, ^4^, ^5^, ^6^ ^]^ However, most of the window‐direct solar energy will be converted into heat in summer.^[^ ^5^, ^7^, ^8^ ^]^ Moreover, the windows will cause 30% of interior energy loss in winter.^[^ ^9^, ^10^, ^11^, ^12^ ^]^ Most of the studies on energy‐saving windows are focused on chromogenic technologies, including thermotropic,^[^ ^10^, ^11^, ^13^, ^14^ ^]^ electrochromic^[^ ^11^, ^15^, ^16^ ^]^ as well as photochromic,^[^ ^12^, ^17^, ^18^ ^]^ which can change the transmittance of color in response to external stimuli such as heat, electricity, and light.^[^ ^6^, ^8^, ^19^, ^20^, ^21^, ^22^ ^]^ Among three, thermotropic smart windows with cost‐effective, rational stimulus and zero energy input properties, which endowed the thermotropic materials including hydrogels and liquid crystals become the most popular among the three kinds of materials.^[^ ^20^, ^23^, ^24^, ^25^, ^26^, ^27^ ^]^ However, the original hydrogel smart windows can only achieve the function of adjusting sunlight transmittance, but cannot meet the more complex needs in practical applications. Hydrogel‐based composites can not only adjust the sunlight transmittance, but also improve the mechanical and thermal response speed of the materials, which endow a better application prospect in smart windows.

Currently, smart windows mainly pay attention to regulating the ability of light transmission. However, in the process of sunlight regulation, the energy of solar radiation is wasted.^[^ ^28^, ^29^ ^]^ As clean and abundant energy, solar energy is expected to alleviate the current global energy crisis.^[^ ^30^, ^31^, ^32^ ^]^ Based on this perspective, it is an attractive research direction to combine energy saving and energy storage in one device.^[^ ^26^, ^27^, ^33^, ^34^, ^35^, ^36^ ^]^ It has been proved that integrating solar cells with VO_2_ thermotropic layers or thermotropic perovskite materials can realize two characteristics.^[^ ^21^, ^37^ ^]^

Zhou et al.^[^ ^38^ ^]^ designed a smart window, which combined the VO_2_ nanoparticles with the solar cells. The light scattering induced by the VO_2_ nanoparticles can be utilized by the solar cells with a 7.5% solar modulating ability (Δ*T*_sol_) and a 0.52% photoelectrical conversion efficiency. Yang et al.^[^ ^39^ ^]^ demonstrate a thermotropic solar cell that depended on halide perovskite cesium lead iodide/bromide with a 7% photoelectric conversion efficiency (PCE). The phase transition temperature is 105 °C. The recent results have exposed some problems such as low conversion efficiency of solar energy, complex equipment manufacturing, harsh phase‐transition conditions, and high cost limited the application in the smart window field.

Therefore, the design of an energy saving and energy storage integrated smart window with a solar energy collection system and temperature control adjustment function will realize the effective utilization of solar energy.^[^ ^36^, ^40^, ^41^, ^42^, ^43^, ^44^ ^]^ Among the photoelectric conversion materials, Si‐based solar cells have accounted for more than 70% of the solar power market.^[^ ^41^, ^42^, ^43^ ^]^ However, the energy storage and energy‐saving integration smart window that integrates the Si‐based solar cells and energy‐saving traditional smart windows have not been reported so far.^[^ ^43^, ^44^, ^45^, ^46^ ^]^

**Figure 1:**
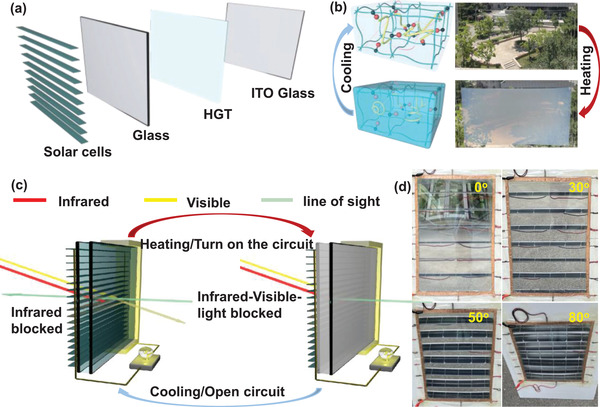
a) The multi‐layer louver structures of the energy saving and energy storage integrated smart window; b) the mechanism schematic of the host‐guest thermochromic hydrogel (HGT hydrogel) and the optical photos for 0.15 m^2^ scale window with the 50 × 30 cm in length testing at a different temperature; c) the schematic diagram of the energy saving and energy storage integrated smart window with the different environments; and d) the optical photos of HGT hydrogel smart window‐based house with the different visual angle.

Hereby, we developed a revolutionary smart window with a multi‐layer louver structure that contains a silicon solar cell, thermotropic hydrogel, and ITO active layer as shown in **Figure** **1**a, which combined both an energy saving and energy generation ability (ESEG smart window) with leverages high solar energy modulation together with high PCE. The energy saving performance of the ESEG smart window was depended on the thermotropic property of the host‐guest thermotropic hydrogel (HGT hydrogel). The phase transition temperature of the HGT hydrogel was ≈45 °C,^[^ ^47^ ^]^ effectively resulting in a reduction of the solar energy entering at high temperatures. The solar modulating ability (Δ*T*_sol_) of the HGT hydrogel was 53.87%, with a high luminous transmittance at 88.68%, which indicated this hydrogel is promising for energy saving smart window applications. The PCE of this ESEG smart window was 18.24% with long‐time stability, as the PCE of most previous reported energy saving and generation integrated smart windows is less than 10%.^[^ ^8^, ^13^, ^39^, ^48^, ^49^ ^]^ In addition, the energy generated by silicon solar cells can realize the automatic adjustment of window temperature and active control depended on the ITO layer, effectively avoiding the problem of window freezing under low temperature. The novel multi‐layer louver structure of the energy saving and energy storage integrated smart windows show an enlightening concept, which may revolutionize the window industry and lead to a new perspective to design the smart window.

## 2 Results

Figure 1b shows the mechanism schematic of the host‐guest thermochromic hydrogel (HGT hydrogel) with different temperatures. The HGT hydrogel is fabricated by thermo‐responsive hydroxypropyl cellulose (HPC) microparticles within a transparent and thermal stable (PAM‐PAA) hydrogel matrix. As shown in Figure 1b, below its lower critical solution temperature (LCST), HPC is well distributed in the PAM‐PAA matrix, and forms hydrogen bonding with the surrounding water molecules,^[^ ^47^ ^]^ the HGT hydrogel is transparent at the moment. However, with the temperature increased to above the LCST (Figure 1b), the interactions and the hydrogen bonding and intramolecular interactions of HPC changed, and the HPC molecules will self‐associated into metastable nanosphere without precipitation, which will cause scattering of the light (Figure S1, Supporting Information) and the HGT hydrogel will turn opaque.^[^ ^47^, ^50^ ^]^ Figure S2, Supporting Information, shows the SEM EDS mappings of the HGT hydrogel at different temperatures. The PAM‐PAA framework structure was stable during the temperature changing process, which endows the long‐time stability of the smart windows. To exhibit the multi‐layer louver structures of ESEG smart windows, we fabricated a smart window as shown in Figure 1c. The schematic and multi‐layer louver structure of transmittance active modulation of this paper is shown in Figure 1c. The images represent the behavior of hybrid smart windows at 25 (left) and 80 °C (right), respectively. The near‐IR from the sun enters the Si‐based solar cells and is converted into electricity. The visible light passes through the sandwich structure at low temperatures. Moreover, the temperature increasing of the glasses due to the long‐time sun exposure leads to the microstructure change of thermotropic hybrid hydrogel and decrease the transmittance. Furthermore, the window can proactively change the transparency at low temperature, which benefits the application of ITO conductive glass. The optical images from different viewpoints are shown in Figure 1d, proving that the visual effect is not affected when the line of sight is at an angle of 90–60° to the window.

**Figure 2:**
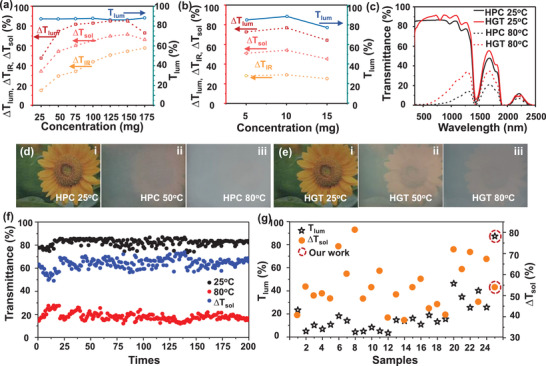
a) Transmittance spectra of optimal HPC concentration and optimal HGT hydrogel at 25 and 80 °C; b) the optical performance comparison on the luminous transmittance (*T*_lum_) at 25 °C, luminous transmittance difference, IR transmittance (Δ*T*_IR_) difference, and solar modulating ability (Δ*T*_sol_) for the different concentrations; c) the optical performance comparison on the luminous transmittance (*T*_lum_, 25 °C) at 25 °C, luminous transmittance difference (Δ*T*_lum_), IR transmittance difference (Δ*T*_IR_), and solar modulating ability (Δ*T*_sol_) for the different concentrations; d) optical photos for pure HPC and e) HGT hydrogel at 25, 50, and 80 °C, respectively; f) cycling testing for the HGT hydrogel smart window; and g) comparison of this work with the other work^[^ ^16^, ^25^, ^26^, ^33^, ^34^, ^35^, ^51^, ^52^, ^53^, ^54^, ^55^, ^56^, ^57^, ^58^, ^59^, ^60^, ^61^, ^62^, ^63^, ^64^ ^]^ regarding the *T*_lum_ and Δ*T*_sol_.

The transmittance spectra of the HPC solution and HGT hydrogel with the different concentrations at both 20 and 80 °C are shown in Figures S3 and S4, Supporting Information. All the samples show a high luminous transmittance (*T*_lum_) at room temperature. **Figure** **2**a,b summarizes the optical properties for different concentrations samples. It can be observed that the transmittance modulation abilities for luminous (Δ*T*_IR_, 87.27%), IR and solar wavelength are all increasing with the increase of HPC and PAA concentrations. The transmittance modulation abilities of luminous, IR, and solar wavelength were increasing gradually and then decreasing with the increasing concentration of HPC. As the 15 mg/6 mL sample provided both the largest luminous transmittance (Δ*T*_lum_, 87.27%) and solar modulating ability (Δ*T*_sol_, 80.11%) (Figure S5 and Table S1, Supporting Information), the 15 mg/6 mL HPC concentration was selected to design the HGT hydrogel. As shown in Figure 2b, compared with other samples, the 10 mg/6 mL PAA HGT hydrogel was selected to fabricate the HGT hydrogel smart window, as it provided the highest *T*_lum_ (88.68%) at room temperature and largest Δ*T*_sol_ (54.02%) (Figure S6 and Table S2, Supporting Information). The results demonstrated that the novel macromolecular polymers are a promising thermotropic material. Figure 2c shows the transmittance spectra of the selected samples with concentrations of 15 mg/6 mL HPC and 10 mg/6 mL HGT hydrogel at different temperatures, respectively. Transmittance hysteresis loop at 600 nm were recorded during the heating and cooling cycles at temperatures ranging from 20 to 60 °C for the 0.1 mm thickness samples as shown in Figure S7(a), Supporting Information. Figure S7(b), Supporting Information, shows the derivative graph of the LCST of HGT hydrogel compared with the pure HPC, which was used to calculate the average LCST. The average LCST of the HGT hydrogel was ≈30 °C, which is lower than the pure HPC (≈45 °C). This is probably due to the introduction of PAA can change the hydrogel structure, which lowers the LCST of the HGT hydrogel. It is worth mentioning that the absorption peak at 1400 and 1900 nm are due to the water molecule's vibration in the HGT hydrogel. The optical photos of the HPC and HGT hydrogel samples are shown in Figured 2d and 2e at 25, 50 as well as 80 °C. In addition, the temperature rising rate curves of the HGT hydrogel with different voltages are shown in Figure S8, Supporting Information. The optical photos (Figure S9, Supporting Information) show the transmittance change after turn on the voltage, demonstrating the existent of ITO endows the HGT hydrogel an active control function. The two samples were transparent at a low temperature, which agrees with the spectra. However, the luminous transmittance decreased dramatically under the high temperature of the two samples. In contrast, the pattern under the HGT hydrogel sample is invisible and misty at 80 and 50 °C, respectively. Thus, the thermochromic optical properties of the HGT hydrogel were regulated by changing temperature. Then we tested the antifreeze effect of our structure (Figure S10, Supporting Information); tested the icing condition of two smart window structures without and with load voltage (10 V) at −5 °C. It can be seen that at 30 min, the smart window without current has been deformed. The smart window we designed and prepared under 10 V load voltage can still show a good working state after 2 h, which proves that it has a very good anti freezing effect.

The cycling stability of the HGT hydrogel smart window is shown in Figure 2f, the change of transmittance of the HGT hydrogel smart window at 650 nm at both 25 and 80 °C is negligible after 200 cycles. The microscopic morphology and thermochromic optical properties were still not changed after the cyclic test, as shown in the SEM images (Figure S11, Supporting Information) and optical photos (Figure S12, Supporting Information) results. Compared with the other reported works,^[^ ^16^, ^33^, ^46^, ^57^ ^]^ the method that we provide in this work achieves a higher *T*_lum_ and a satisfactory Δ*T*_sol_ as shown in Figure 2g, which confirmed the superiority of the HGT hydrogel. The characteristic peaks of different samples were presented by the Fourier transform infrared spectroscopy (FTIR) in Figure S12, Supporting Information.

**Figure 3:**
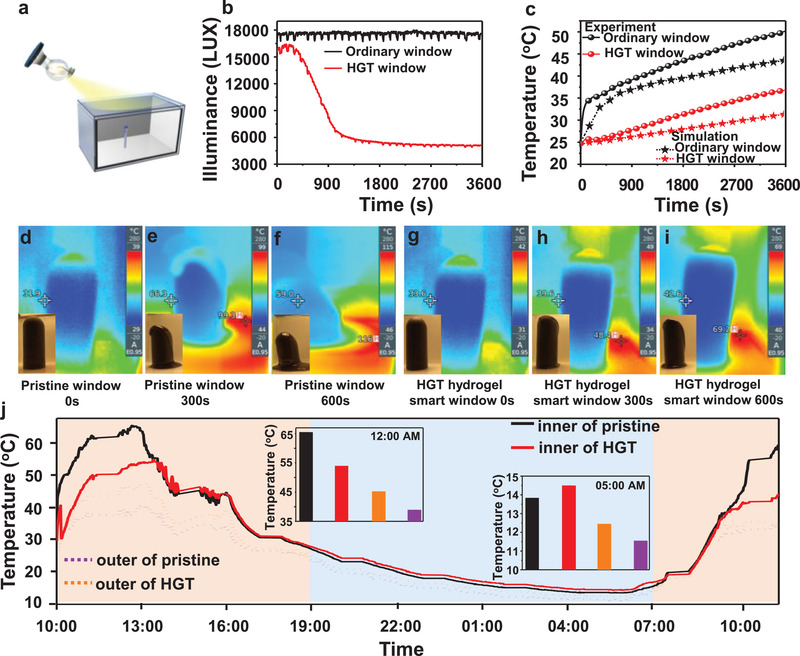
a) Scheme of the indoor thermal and illumination intensity test set up for ordinary and HGT hydrogel smart window; b) the indoor illumination intensity with the ordinary window and HGT hydrogel smart window with the simulated sunlight; c) the indoor temperature with the reference and HGT hydrogel smart window under the simulated sunlight; The thermal infrared images and optical photos of the ordinary window‐based house under the simulated sunlight at d) 0, e) 300, and f) 600 s and HGT hydrogel smart window‐based house at g) 0, h) 300, and i) 600 s; j) 24 h air temperature curve for the outdoor demonstration in Beijing. The inserts are the daytime (12:00) and night (05:00) temperature readings for the pristine and HGT hydrogel window‐based house, respectively.

Based on the discussion above, the HGT hydrogel smart windows possess a satisficing light regulating ability. The indoor thermal test was designed to explore the solar modulation and energy‐saving performance of HGT hydrogel. **Figure** **3**a shows the illustration of the experimental setup for the indoor thermal test. The device was installed onto the glasshouse (30 cm × 20 cm × 20 cm) to study the indoor illumination intensity and temperature change. The indoor illumination intensity with the pristine window and HGT hydrogel smart window was evaluated with the simulated sunlight by the luminometer as shown in Figure 3b and the experiment set up is shown in Figure S14, Supporting Information. When the environment temperature is above the LCST, the transmittance dropped dramatically to block the solar light.

Figure S15, Supporting Information, shows the temperature experiment set up and Figure 3c shows the curves of indoor temperature which is based on the HGT hydrogel smart window and the pristine under the simulated sunlight, verified at a 13.3 °C lower indoor temperature than the pristine house after 1 h radiation. However, the external surface temperatures of the pristine windows and HGT hydrogel smart window were 42.1 and 63.7 °C as shown in Figure S16, Supporting Information. It is similar to the experimental results (Figure S18) in which the temperature grows slowly in the HGT hydrogel smart window‐based house than the pristine, demonstrating an excellent energy‐saving performance of the HGT hydrogel window. The photos of outdoor devices and the test results in Beijing are shown in Figures S17 and S18, Supporting Information, which is similar to the simulated sunlight results. The indoor and external surface temperatures (Figure S19, Supporting Information) can fluctuate by as much as 6.7° and 7.6° after 1 h of sunlight, respectively.

To further demonstrate the energy saving performance of the HGT hydrogel window, the chocolates were placed inside the house model to evaluate the energy‐saving effect of the different windows as shown in Videos S1 and S2, Supporting Information. The changes in object temperatures and morphology were recorded by the thermal infrared and optical camera as shown in Figure 3d–i. It can be found that the chocolate temperature in the HGT hydrogel‐based smart window house was lower than the pristine with the same illumination time, indicating the HGT hydrogel smart window has an excellent energy saving and energy storage function. The chocolate was melted after 300 s of illumination in the reference house (Figure 3h), while the chocolate in HGT hydrogel‐based smart window house has no obvious changes (Figure 3e). After 600 s of illumination, as in Figure 3i, the chocolate temperature rises to 42.6 °C. However, the temperature of chocolate reached 59.0 °C at the same time (Figure 3f). Figure 3j shows the experiment results under the hot environment in Beijing. At noon 1:00 PM, the highest house air temperature is 65.7 °C based on the pristine glass window. And the HGT hydrogel smart window‐based house is 54.9 °C at 1:30 PM, which is preferred to shift electricity usage to low utility price periods.

To further analyze the temperature distribution in the house, and energy‐saving simulation was used to calculate the energy‐saving performance of the HGT hydrogel smart window in the house, as shown in Figures S20–S22, Supporting Information. The simulation temperature distribution diagram of the reference and HGT hydrogel‐based smart window house with the main view and profile at 12:00 AM are shown in Figure S20, Supporting Information. HGT hydrogel‐based smart window house has a lower indoor temperature in the hot environment compared with the pristine, which is benefited by the reduced Δ*T*_sol_ (54.02%) of the HGT hydrogel smart window compared with the reference (8.0%). Similar results of the temperature distribution at 9:00 AM and 3:00 PM are shown in Figures S21 and S22, Supporting Information, respectively.

**Figure 4:**
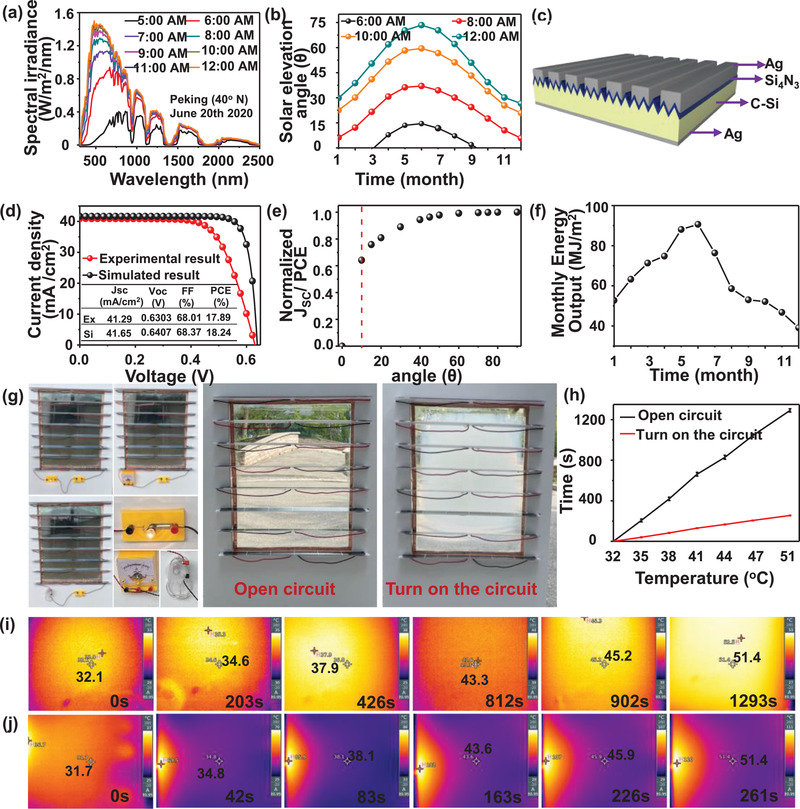
a) Solar spectral irradiance varying with time on June 20, 2020, in Beijing; b) monthly solar elevation angle with a different time in Beijing; c) schematic of the solar cell model; d) *J*–*V* curves measured and simulated on c‐Si solar cells; e) normalized *J*_sc_/PCE of c‐Si solar cell varying with incident angle; f) the monthly energy output of c‐Si solar cell in Beijing; g) the optical photos of HGT hydrogel smart window with the different functions; and h) the temperature rise rate curves of windows with different voltages; the thermal infrared images with the different time under 0 (i) and 16 V (j) voltages.

To evaluate the influence of varying times on energy released by the sun, we have calculated *J*_sc_ and PCE based on crystalline silicon (c‐Si) solar cells under real solar spectra. The city (Beijing, latitude ≈ 40°N and longitude ≈ 116°E) was chosen with the real simulated solar spectra under clear‐sky conditions at different dates and times obtained from the website PVlighthouse.com. **Figure** **4**a shows the solar spectral irradiance with time ranging from 5 to 12 o'clock on June 20, 2020, in Beijing. We took the maximum point at each mouth respectively and plotted the solar irradiance varying with time, as shown in Figure 4b. The performance of the c‐Si solar cell purchased on the market was measured, and then the cell was installed vertically on the smart window. The solar cell model is constructed according to the simulation of the real solar cell using Comsol Multiphysics software 5.3, which consists of a 100 nm thick Si_3_N_4_ passivation layer, 200 µm thick c‐Si with pyramid textured surfaces (6 µm), and 300 nm thick Ag back electrode as shown in Figure 4c. The measured and simulated *J*–*V* curve is obtained in Figure 4d. In the simulation, we ignored the series resistance and parallel resistance of the c‐Si solar cell, which caused the error of the measured result and the experimental result. Thereafter, the normalized *J*_sc_ changes at different incident angles are simulated and exhibited in Figure 4e.

The monthly energy output E has been calculated by Equation (1).

(1) $$E\;=\int P_{\mathrm{illum}}\left(t\right)\;\times \;t\times \;PCE\left(t,\theta \right)dt$$

Where *P*_illum_(*t*) is average solar irradiance in a certain hour obtained from the website Energyplus.com. And PCE (*t, θ*) is the photoelectrical conversion efficiency of the c‐Si solar cell at different times (*t*). According to the solar elevation angle (*θ*) at different times, the corresponding PCE can be found in Figure 4f. Therefore, the energy output can be easily obtained versus time during 1 day, which is detailedly listed in Table S3, Supporting Information. As shown in Figure 4e, the monthly energy output of the c‐Si solar cell in Beijing increases over time, reaching a maximum of 90.64 MJ m^−2^ in June, and then continuously decreasing, reaching a minimum of 39.09 MJ m^−2^ in December. The annual energy output of the c‐Si solar cell in 2020 is 766.74 MJ m^−2^. It is worth noting that the reported maximum PCE of the c‐Si solar cell is 26.3%,^[^ ^41^ ^]^ which is 18.24% of what we used here. Therefore, the annual energy output has the potential to reach 1105.55 MJ m^−2^. The optical images of the ESEG smart window are shown in Figure 4g. Under the condition of the single‐chip solar panel used as the power supply, the display of the ammeter was 2 A, the bulb of 1.5 V teaching experiment could emit bright light, and the electric fan with the rated voltage of 2 V worked normally. It can be found that the electrical appliances work well with the self‐powered system. To prove the smart window can realize active control functions under the sunshine, the ESEG smart window was placed outside in a sunny place. As a result, the temperature rise rate of the ESEG smart window under 16 V (all the panels are connected in series [Figure 4g] the voltage was five times more than the 0 V as shown in Figure 4h. The thermal infrared images with the same temperature of under the 0 and 16 V voltage are shown in Figures 4i and 4j, demonstrating the ESEG smart window possessed autonomic regulation. In addition, the ESEG smart window has the function of antifreezing, which prevents the risk of water‐based windows freezing and cracking at low temperatures.

## 3 Conclusion

A disruptive novel smart window (ESEG smart window) integrating the high solar modulation of host‐guest thermochromic hydrogel (HGT hydrogel) and energy generation of solar cells was designed and prepared in this paper. The Δ*T*_sol_ of the ESEG smart window was 54.02%, which depended on the HGT hydrogel. The PCE of the silicon solar cell was 18.24%, with long‐time stability at 25 °C under 40–60% humidity, endowing a satisficing energy storage ability. In addition, the ITO was introduced as resistance, where the energy generated by silicon solar cells can be used to adjust automatically the window temperature and realize active control, avoiding window freezing effectively under low temperature. The device multi‐layer louver structure of the ESEG smart window gives the unique advantages of simple fabrication and scale‐up with an energy storage, energy‐saving, active control, and anti‐freezing integration function, which makes it highly promising for commercialization. The revolutionary technology in this paper possesses the enormous potential to cut down carbon emissions and elevate the sustainability of buildings and greenhouses.

## 4 Experimental Section

Hydroxyproply cellulose (HPC, 99%, Sigma Aldrich Inc.), acrylamide (AM, 99%, Sigma‐Aldrich), N‐N′ methylene bisacrylamide (99%, Sigma‐Aldrich), 2‐hydroxy‐1‐[4‐(2‐hydroxyethyl) phenyl]‐2‐methyl‐1‐propanone (Irgacure 2959, Sigma‐Aldrich), and polyacrylic acid (Mr. = 1800, PAA, 99%, Sigma‐Aldrich) were used directly without further purification. 1‐mm‐thick double‐sided closed‐cell acrylic foam tape (3M VHB tape). The deionized water (DI water) was used as the solvent for all the mixed solutions. 5 mm thick planks were used to make the house model.

### Characterization

The transmittance and reflectance spectra were collected on a UV–vis–NIR spectrophotometer system with the integration sphere attached (AvaSpec‐ULS2048L StarLine Versatile Fiber‐optic spectrometer and AvaSpec‐NIR256‐2.5‐HSC‐EVO, Avantes, the Netherlands). The *T*_lum_, *T*_IR_, and *T*_sol_ were calculated with the references.^[^ ^3^ ^]^

### Preparation of Host‐Guest Thermochromic (HGT) Hydrogel

0.1 g of HPC monomer were dissolved in 6 mL DI water at room temperature to obtain the HPC homogeneous mixed aqueous solution. Subsequently, keeping the mixed solution at 70 °C with a magnetic stirring for 25 min and then the mixed solution was cooled down to room temperature naturally. Then, 1.2 g AM monomer, 12 mg N‐N′ methylene bisacrylamide, 12 mg 2‐hydroxy‐1‐[4‐(2‐hydroxyethyl) phenyl]‐2‐methyl‐1‐propanone as well as 0.15 g PAA were added to the mixed solution. The mixed solution was stirred with the magneton for 24 h.

### Preparation of Host‐Guest Thermochromic Hydrogel Smart Window

The hybrid hydrogel precursor was directly poured into the prepared sandwich glasses structure (one is the glass and another is ITO glass) with 1 mm thickness and then the sprue was sealed with silicone gel. Subsequently, the 1‐mm sandwich hybrid hydrogel smart window precursor was put under the UV‐light environment to obtain the sandwich hybrid hydrogel smart window.

### Preparation of the Energy Saving and Energy Storage Integrated Smart Window

The silicon solar cell was installed on the sandwich hybrid hydrogel smart window and the authors kept the width of the silicon solar cell at 1/5 the length of the glasses. Then, the silicon solar cells were connected in series.

### The Calculation of the *J*_SC_, *V*_oc_, FF, and PCE of the Silicon Solar Cell

Based on the solar spectra and the active absorption spectra, *J*_SC_^[^ ^44^ ^]^ varying with time can be calculated using Equation (2):^[^ ^44^ ^]^

(2) $$J_{\mathrm{SC}}=\frac{q}{hc}\;\int_{300\;\;nm}^{800\;\;nm}\lambda A\left(\lambda \right)\emptyset_{\mathrm{AM}1.5G}\left(\lambda \right)d\lambda$$

The open‐circuit voltage (*V_oc_*)^[^ ^44^ ^]^ calculated from the *J*_sc_ by the Shockley diode Equation (3) is given as:

(3) $$V_{\mathrm{OC}}=\;\frac{k_{B}T}{q}\ln\left(\frac{J_{\mathrm{SC}}+J_{0}}{J_{0}}\right)$$

Where *K*_B_ is the Boltzmann constant and *T* is the room temperature (298 K). *J*_0_ is the diode saturation current density which is set to 6.2 × 10^−10^ mA cm^−2^ here. The fill factor (FF) was calculated through an empirical Equation (4):

(4) $$\mathrm{FF}\;=\frac{V_{\mathrm{OC}}-\frac{k_{B}T}{q}\ln\left[\frac{qV_{\mathrm{OC}}}{k_{B}T}+0.72\right]}{V_{\mathrm{OC}}+\frac{k_{B}T}{q}}\;$$

The PCE of the simulated solar cell was obtained by Equation (4):

(5) $$\mathrm{PCE}=\frac{V_{\mathrm{OC}}\times J_{\mathrm{SC}}\times \mathrm{FF}}{1000\;W\;\;m^{-2}}\;$$

Noted that the value of solar irradiance is lower than 10° (red line left), the solar cell module is set to not work. When the *J*_sc_ does not significantly change, the *V*_oc_ and FF change very little through Equations (3) and (4). Therefore, to simplify the calculation, we set *V*_oc_ and FF to not change with the incident angle, which means that the normalized *J*_sc_ and PCE are consistent.

### Preparation of Samples for HGT Smart Window

The polymeric precursor mixed solution was poured directly into the prepared simple glass box with a 1 mm reserved space to form the sandwich structure (Figure S23, Supporting Information), which was then sealed with silicone gel. Subsequently, the encapsulated HGT smart window was placed in the UV of 365 nm and cured for 1 h.

### Indoor Luminosity and Thermal Test Procedure

Indoor luminosity and thermal testing are proof for the function of regulating solar light and energy saving. This test provides a controlled environment for the experiment without temperature fluctuation and compares the glass panel with the HGT smart window. It can provide an accurate evaluation of energy saving. The indoor lighting testing environment temperature is 25 °C. In addition, the glass box (30 cm × 20 cm × 20 cm) was used as the indoor test equipment which covered five pieces with 2‐cm‐thick Styrofoam. The luminometer and thermocouples were used to detect the illumination intensity and temperature, respectively. The location of the thermocouples at the outside surface and inner surface were used for the temperature of windows and inner air temperature. The 250 W solar light was used as the simulated solar light source, which was placed on the glass box with the angles of 60^o^ and 90^o^ with a 25 cm vertical distance for illumination intensity and temperature test as shown in Figures S13 and S14, Supporting Information, respectively.

### Outdoor Test Procedure

Outdoor thermal testing is proof of the function of energy saving with a realistic environment for the experiment, which compares the glass panel with the HGT smart window. It can provide an actual evaluation of energy saving. The outdoor lighting testing environment temperature is 28 °C with a test glass box (30 cm × 20 cm × 20 cm), which is placed on the playground with enough light as shown in Figure S15, Supporting Information. The glass box was covered in five pieces with 2‐cm‐thick Styrofoam. The thermocouples were used to detect the temperature of the outer window surface and inner air, respectively.

## Conflict of Interest

The authors declare no conflict of interest.

## Author Contributions

These authors (Y.N., Y.Z., and D.D.) contributed equally to the work. Q.X. and Y.Z. designed the experiments. Y.N. did most of the experiments, D.D. did the simulated data, and X.O., F.F., and S.Z. helped with the sample preparation. Y.L. and J.L. helped with the SEM test and analysis. All the authors discussed the results. Y.N. wrote the manuscript. All the authors approved the submission.

## Supporting information

Supporting Information

Supplemental Video 1

Supplemental Video 2

Supplemental Video 3

## Data Availability

The data that support the findings of this study are available from the corresponding author upon reasonable request.
