# Supplementary material for: Energy Saving and Energy Generation Smart Window with Active Control and Antifreezing Functions
Source: Adv Sci (Weinh). 2022 Jan 11;9(6):2105184. doi: 10.1002/advs.202105184 (PMC8867198; doi:10.1002/advs.202105184)
Supplement: Supplementary file 1 — Supporting Information [file ADVS-9-2105184-s004.pdf]

Energy Saving and Energy Generation Smart Window with Active Control and Antifreezing Functions

Yingchun Niu[a=], Yang Zhou[a=]*, Daxue Du[b=], Xiangcheng Ouyang[a], Ziji Yang[a], Wenjie Lan[a*], Fan Fan[a], Sisi Zhao[a], Yinping Liu[a], Siyuan Chen[a], Jiapeng Li[a], Quan Xu[a]*

[a]*State Key Laboratory of Heavy Oil Processing, China University of Petroleum-Beijing, 102249, China*

[b]*School of Environment and Chemical Engineering, Yanshan University, Qinhuangdao 066004, China*

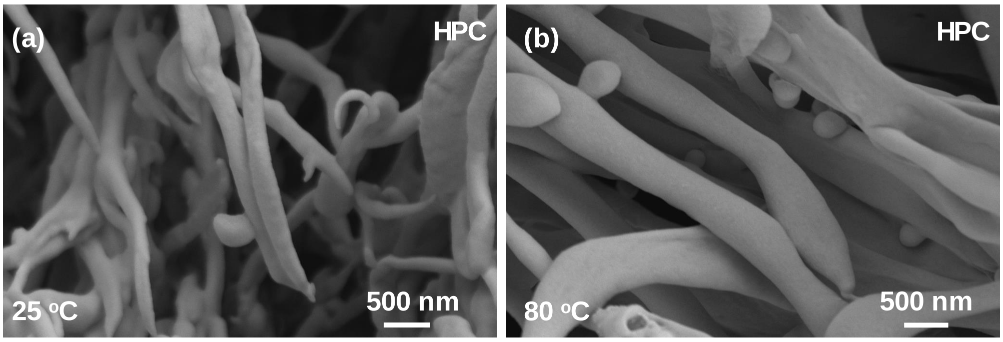

Figure S1 The SEM images of the hydroxypropyl cellulose with the different temperature, a (25 ºC); b (80 ºC).

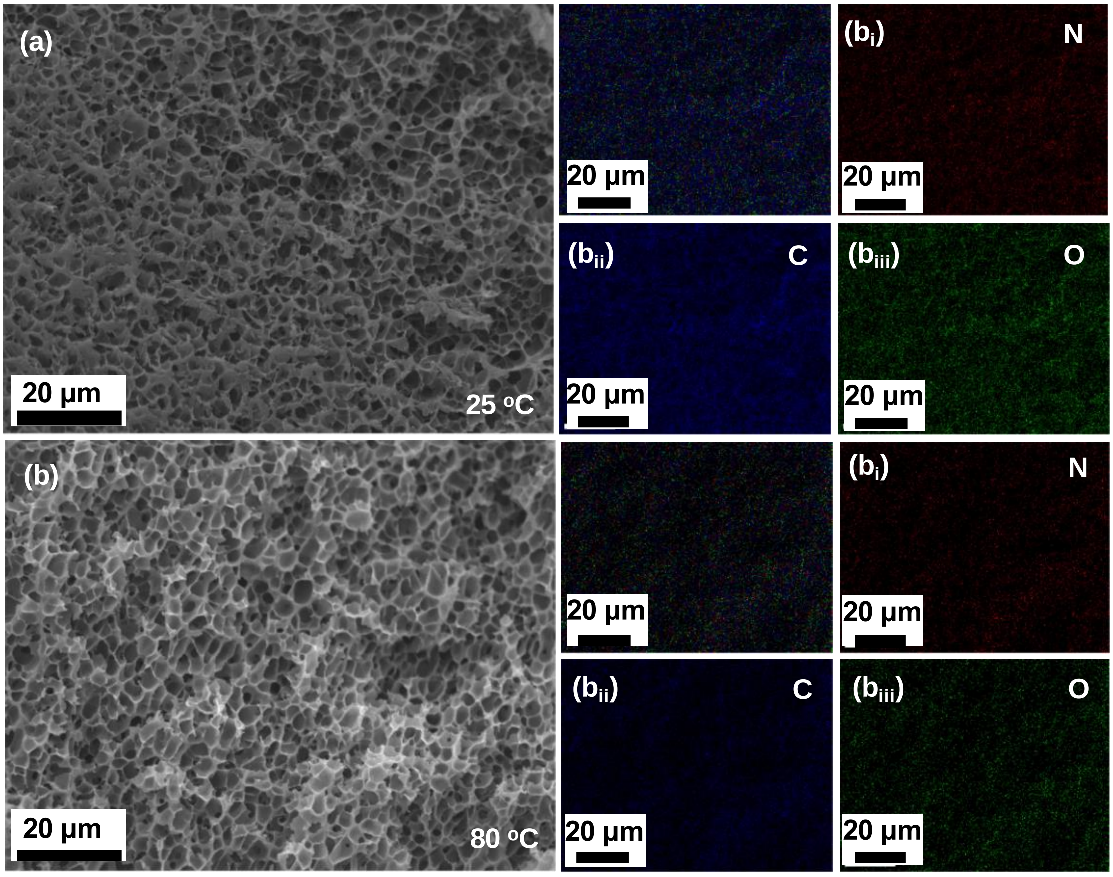

Figure S2 The EDS mappings of the host-guest themochromic hydrogel with the different temperature, a (25 ºC); b (80 ºC).

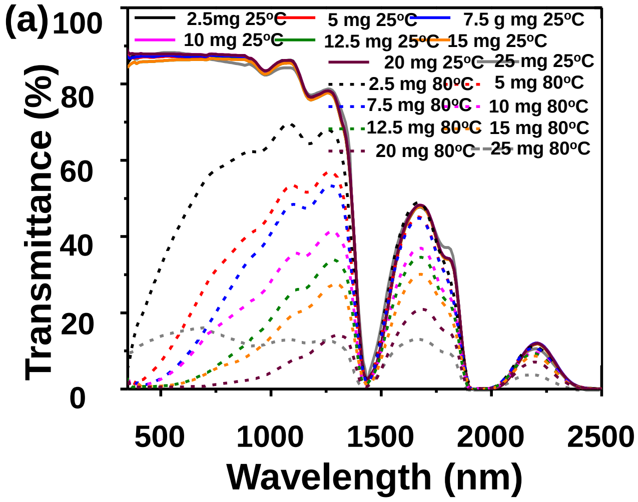

Figure S3 Transmittance spectra of pure HPC with the different concentration.

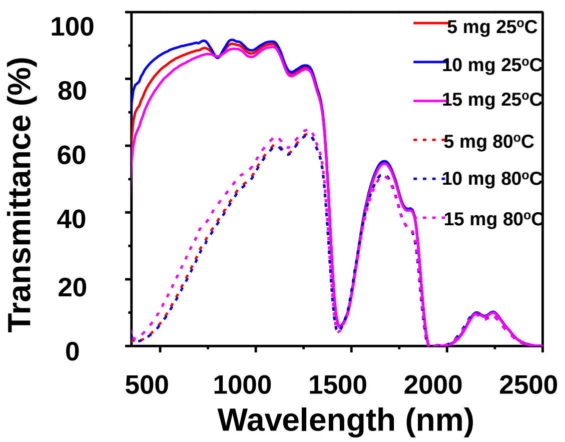

Figure S4 Transmittance spectra of the HGT hydrogel smart window with the different PAA concentration.

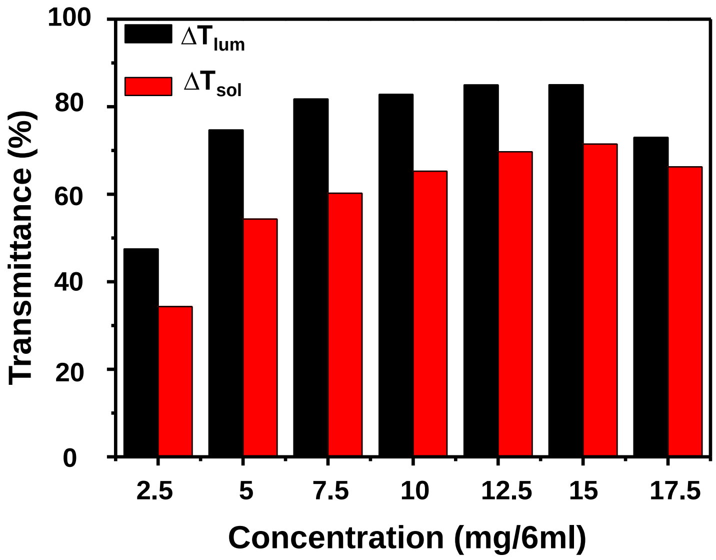

Figure S5 The ΔT_{sol} and ΔT_{lum} values of pure HPC with the different concentration

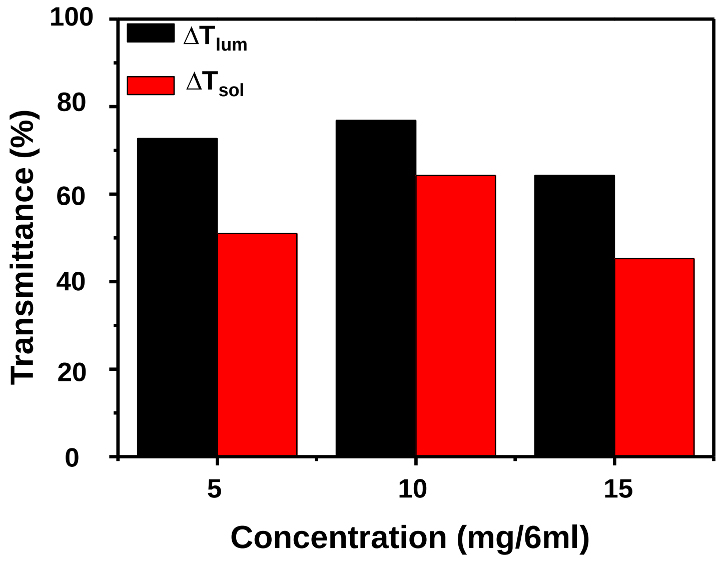

Figure S6 The Δ T_{sol} and Δ T_{lum} values of HGT hydrogel smart window with the different PAA concentration.

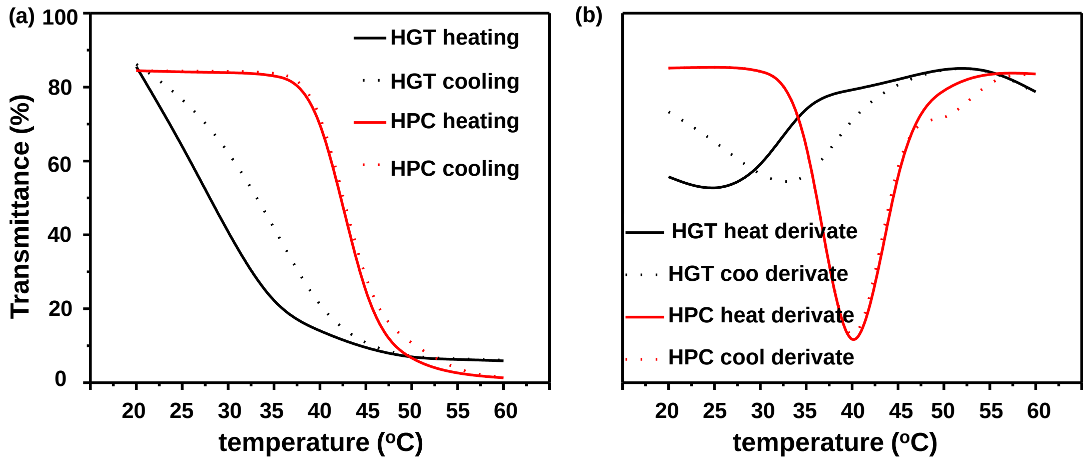

Figure S7 Hysteresis loop (a) and derivative (b) of both HGT hydrogel and pure HPC.

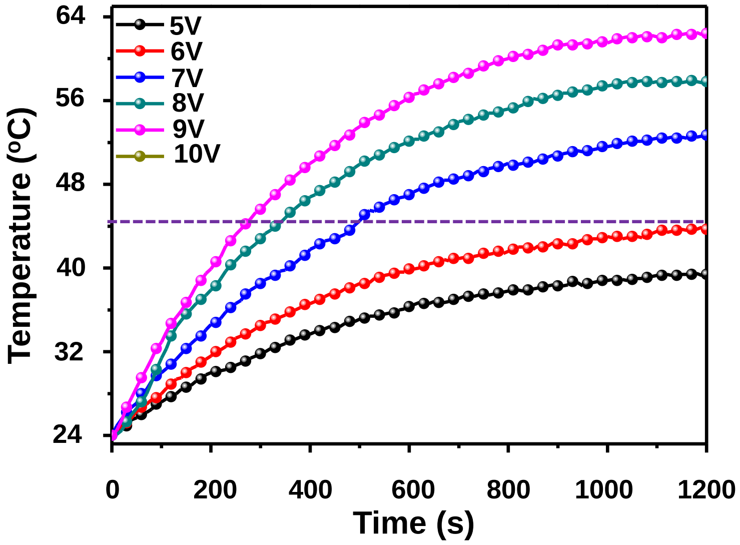

Figure S8 The heating curves of HGT hydrogel window with the different time and voltage.

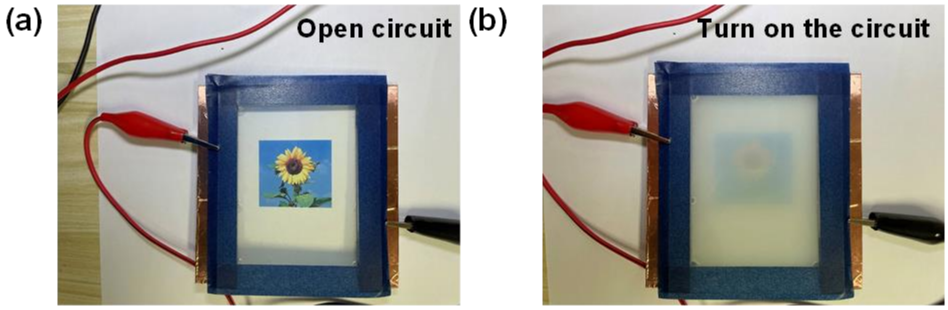

Figure S9 The optical images of HGT hydrogel window at open circuit (a) and turn on the circuit (b).

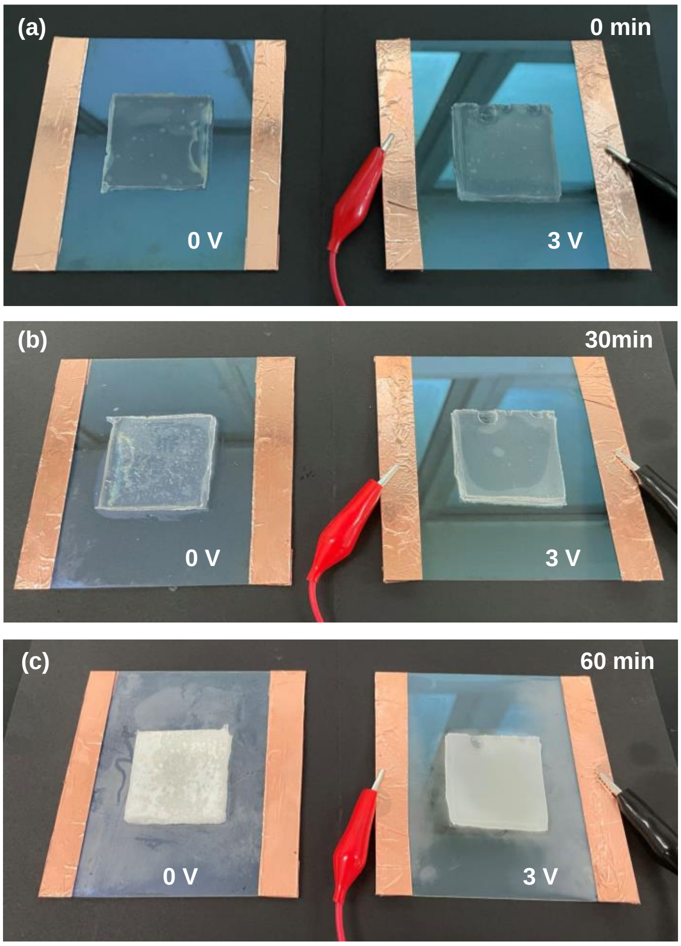

Figure S10 The optical images of HGT hydrogel window under -7 ºC for the different freezing time with and without 3 V loading voltage.

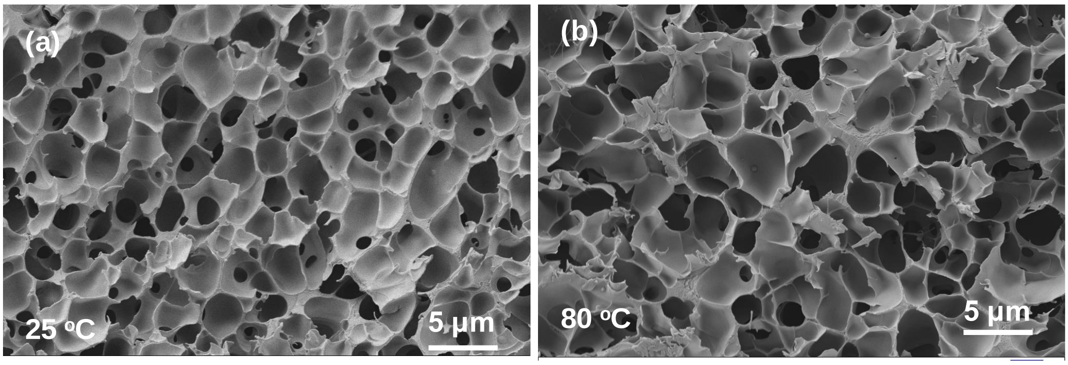

Figure S11 The SEM images of the host-guest themochromic hydrogel with the different temperature, a (25 ºC); b (80 ºC).

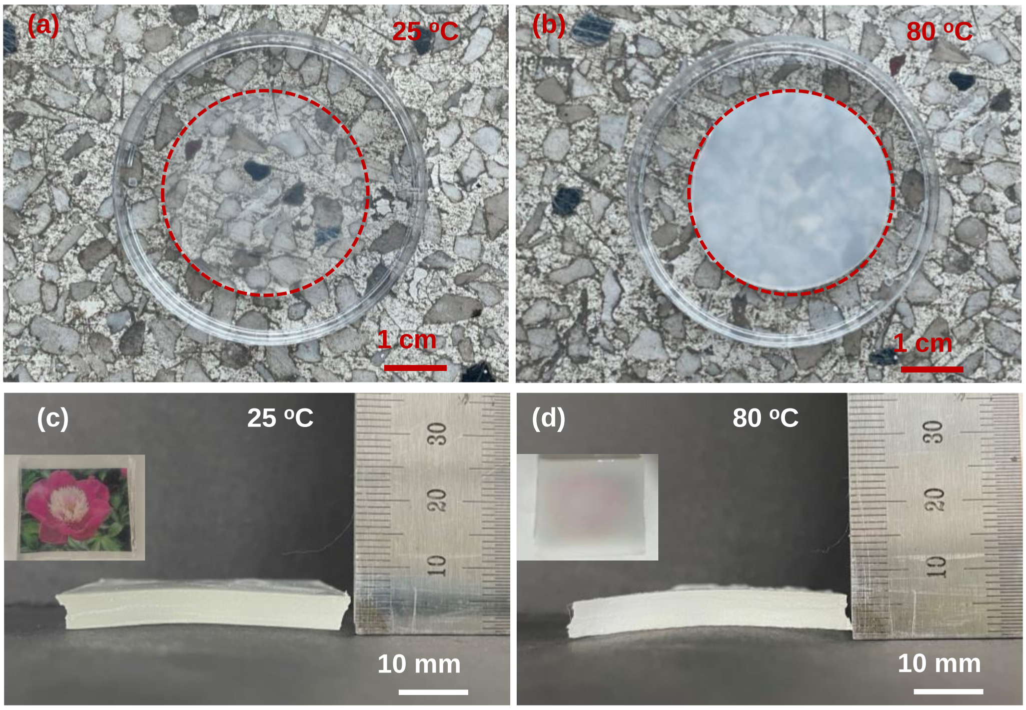

Figure S12 Optical images of host-guest themochromic hydrogel after 200 times cooling (a,c) and 200 times heatin process (b,d).

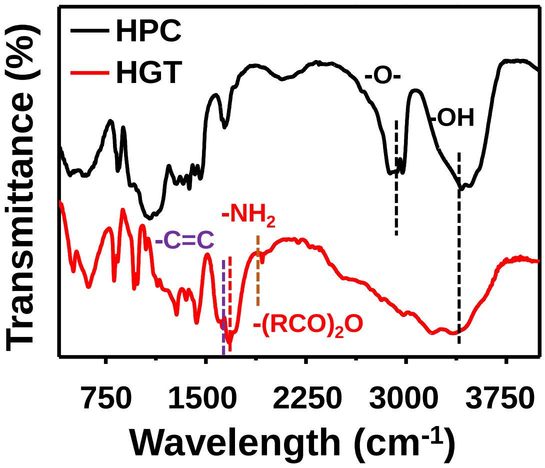

Figure S13 FTIR spectrum for HPC and HGT hydrogel.[1–3]

The characteristic peaks of different samples were presented by the fourier transform infrared spectroscopy (FTIR) in Figure S11, which indicated the changes of functional groups. The distinct absorption peaks at 3440 and 2885 cm^{-1} can be attributed to the vibration of -OH and -O- in the HPC curve.[1–6] The new absorption peaks at 1930, 1669 and 1640 cm^{-1} were attributed to the vibration of $-(RCO)_2O$, $-NH_2$ and -C=C of the HPC curve HGT hydrogel, indicating the formation of HGT hydrogel.[1,2,5,7–9]

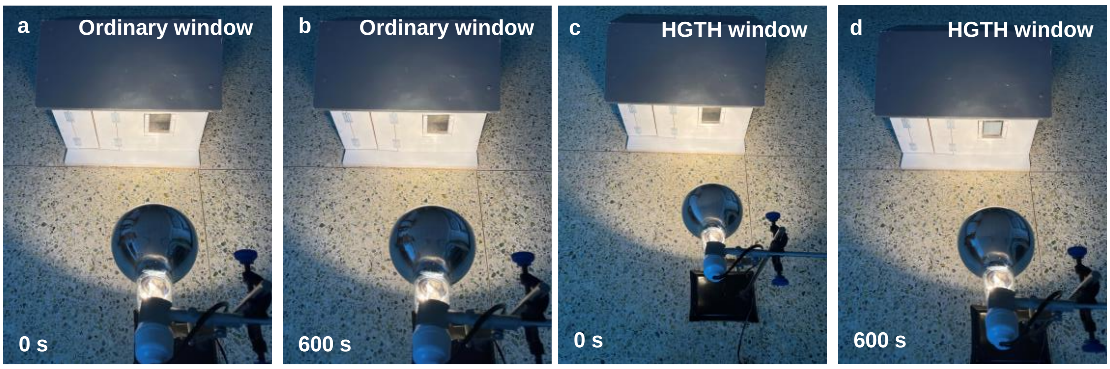

Figure S14 The optical images of the device for the indoor illumination intensity with the pristine window and HGT hydrogel smart window.

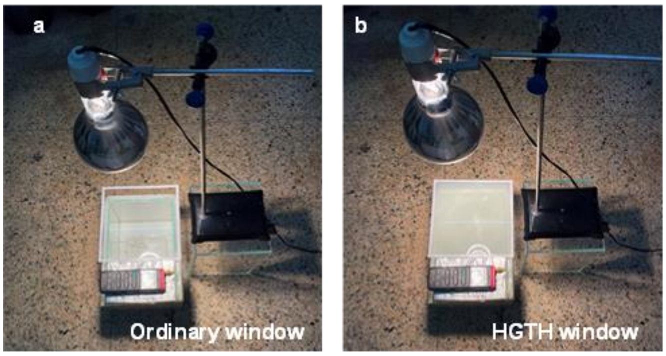

Figure S15 The optical images of the device for the indoor temperature with the pristine window and HGT hydrogel smart window under simulated sunlight.

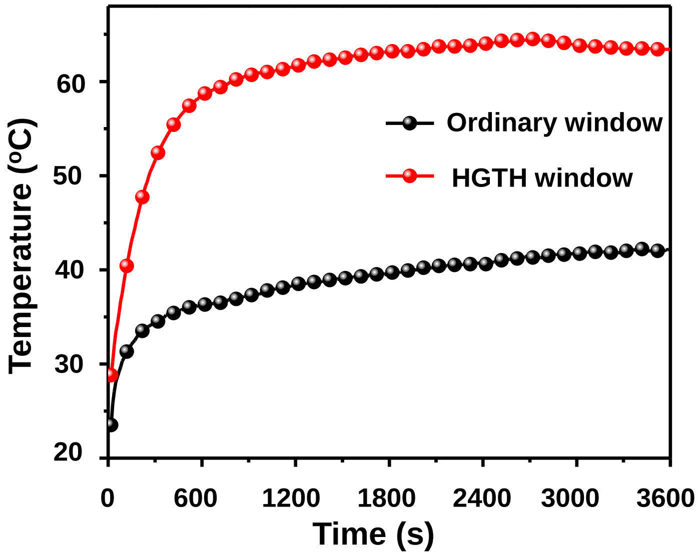

Figure S16 The temperature of outer surface of the windows with the pristine and HGT hydrogel smart window under the simulated sunlight.

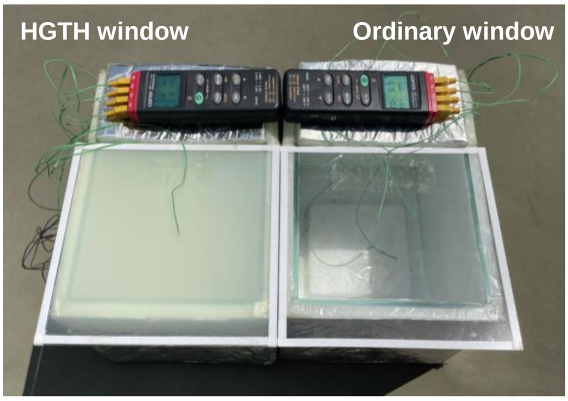

Figure S17 The optical images of the device for the indoor temperature with the pristine and HGTH smart window under sunlight.

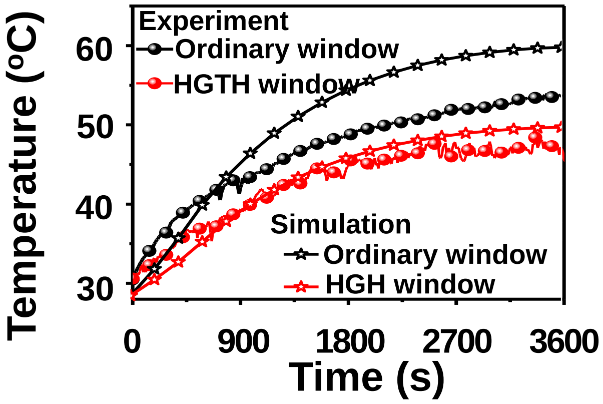

Figure S18 The indoor temperature with the pristine e and HGT hydrogel smart window under the sunlight.

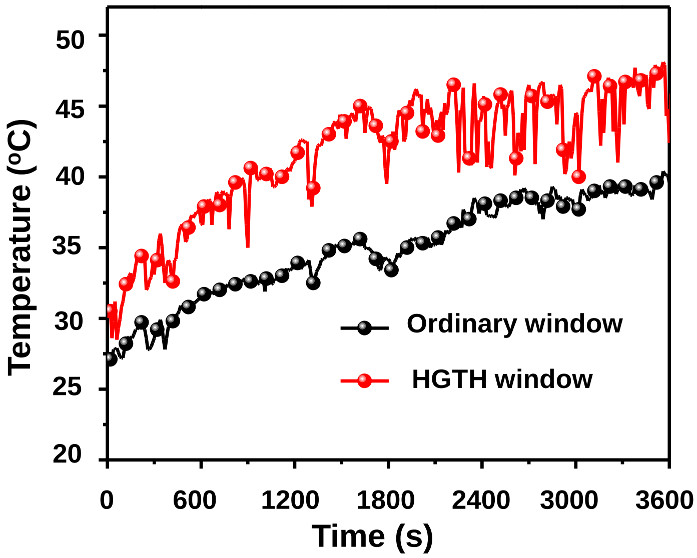

Figure S19 The temperature of outer surface of the windows with the pristine and HGT hydrogel smart window under the sunlight.

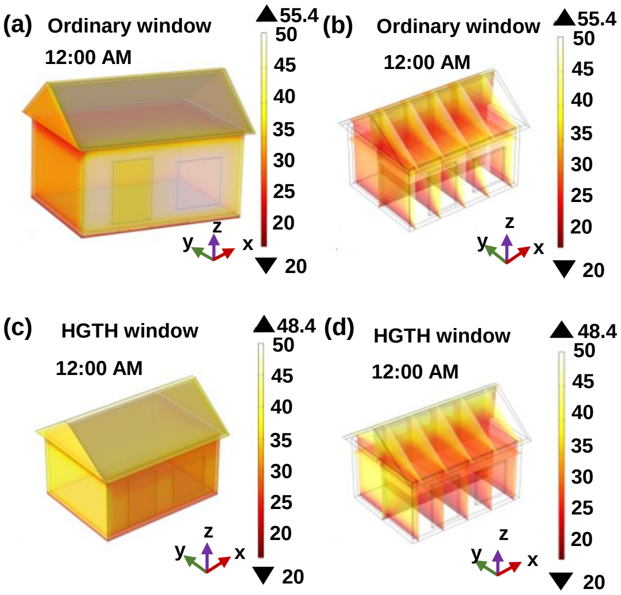

Figure S20 The simulation diagram of indoor temperature distribution of the pristine window-based house (a) main view (b) profile and HGT hydrogel based smart window house (c) main view (d) profile at 12:00 AM.

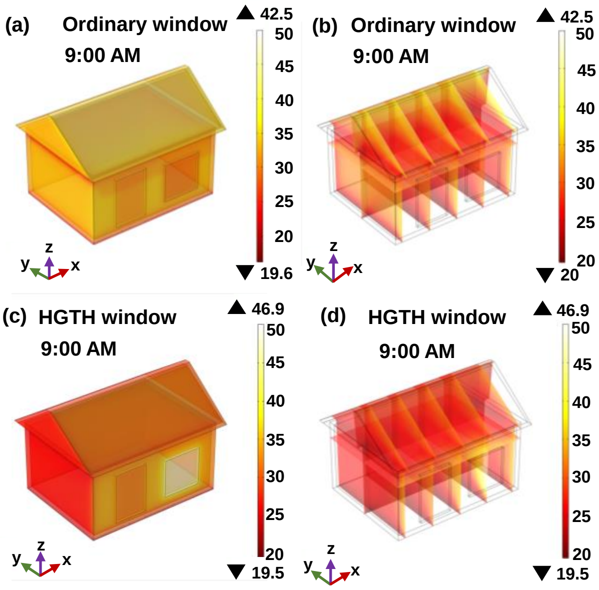

Figure S21 The simulation diagram of indoor temperature distribution of the pristine window-based house (a) main view (b) profile and HGT hydrogel based smart window house (c) main view (d) profile at 9:00 AM.

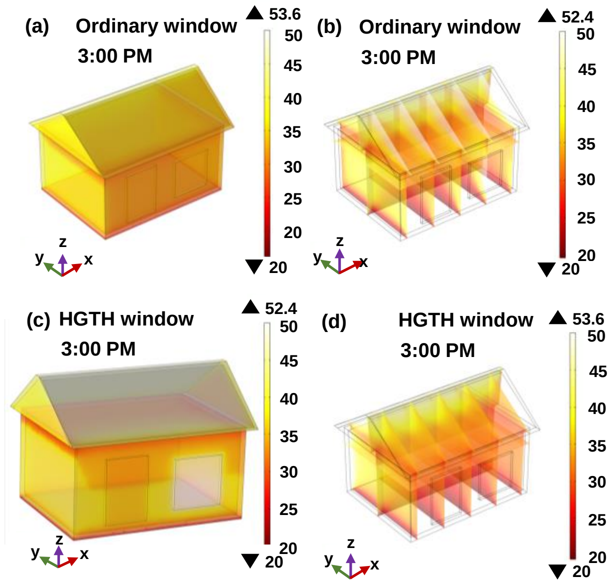

Figure S22 The simulation diagram of indoor temperature distribution of the pristine window-based house (a) main view (b) profile and HGT hydrogel based smart window house (c) main view (d) profile at 3:00 PM.

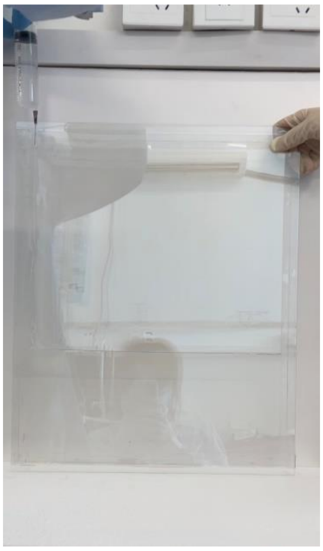

Figure S23 Optical images for the preparationof HGT hydrogel smart window.

Table S1 The values of all the parameters of the HPC with different concentrations.

Concentration	2.5mg	5.0 mg	7.5 mg	10 mg	12.5 mg	15 mg	175 mg
T_{lum} (%)	87.29	87.13	87.36	87.85	86.27	86.27	88.25
ΔT_{lum}(%)	47.51	74.66	81.74	82.83	84.96	85.00	72.97
ΔT_{sol}(%)	34.30	54.34	60.24	65.24	69.72	71.49	66.26
ΔT_{IR}(%)	15.05	29.46	34.22	43.85	50.65	54.52	57.73

Table S2 hydrogel with different concentrations.

Concentration	5 mg	10 mg	15 mg
T_{lum} (%)	85.1%	88.68%	77.25%
ΔT_{lum} (%)	72.67%	76.84%	64.26%
ΔT_{sol}(%)	50.99%	54.02%	45.28%
ΔT_{IR} (%)	28.12%	29.1%	25.2%

Table s3. The energy output of c-Si solar cells versus time during one day in 2020

Time	Energy output (MJ/m^2)											
	Jan	Feb	Mar	Apr	May	Jun	Jul	Aug	Sep	Oct	Nov	Dec
6:00-	0	0	0	0	0	2.84	2.09	0	0	0	0	0
7:00-	0	0	2.11	2.96	4.28	4.43	3.58	2.66	1.93	0	0	0
8:00-	0	4.67	4.47	5.41	5.87	5.41	4.72	3.28	3.33	2.95	1.83	0
9:00-	3.87	8.11	5.81	6.55	6.98	7.45	5.96	5.68	4.76	4.56	4.11	4.14
10:00-	7.36	8.62	6.21	9.22	8.91	7.35	6.65	5.03	5.80	6.37	5.80	6.12
11:00-	7.90	10.24	8.48	6.68	10.20	9.06	6.98	4.91	6.00	5.93	8.31	5.49
12:00-	8.84	12.05	8.83	7.37	9.48	9.06	7.86	7.09	6.24	7.73	7.69	6.67
13:00-	9.04	9.58	9.22	7.95	9.08	9.47	7.44	5.84	6.43	8.10	9.58	6.73
14:00-	8.32	8.07	7.50	10.31	8.44	8.79	7.57	7.05	6.22	6.59	5.41	6.81
15:00-	6.01	6.32	9.01	8.32	9.76	9.68	7.38	8.09	6.59	6.51	5.58	3.19
16:00-	1.43	0	7.42	7.41	7.74	10.27	6.23	4.74	5.65	3.39	0	0
17:00-	0	0	2.27	5.09	7.33	8.31	6.29	4.23	1.80	0	0	0
18:00-19:00	0	0	0	0	0	4.38	3.63	0	0	0	0	0

Reference

[1] M. K. Dietrich, B. G. Kramm, M. Becker, B. K. Meyer, A. Polity, P. J. Klar, *J. Appl. Phys* **2015**, *117*, 185301.

[2] M. K. Dietrich, F. Kuhl, A. Polity, P. J. Klar, *Appl. Phys. Lett.* **2017**, *110*, 141907.

[3] C. Liu, Y. Long, S. Magdassi, D. Mandler, *Nanoscale* **2017**, *9*, 485.

[4] C. Chen, Y. Wang, Q. Wu, Z. Wan, D. Li, Y. Jin, *Chemical Engineering Journal* **2020**, *400*, 125876.

[5] H.-P. Cong, P. Wang, S.-H. Yu, *Small* **2014**, *10*, 448.

[6] D. Wang, H. Li, Z. Liu, Z. Tang, G. Liang, F. Mo, Q. Yang, L. Ma, C. Zhi, *Small* **2018**, *14*, 1803978.

[7] H. Li, Z. Liu, G. Liang, Y. Huang, Y. Huang, M. Zhu, Z. Pei, Q. Xue, Z. Tang, Y. Wang, B. Li, C.

Zhi, *ACS Nano* **2018**, *12*, 3140.

[8] Y. Zhou, C. Wan, Y. Yang, H. Yang, S. Wang, Z. Dai, K. Ji, H. Jiang, X. Chen, Y. Long, *Adv. Funct. Mater.* **2019**, *29*, 1806220.

[9] M. A. Green, *Sol. Cells* **1982**, *7*, 337.
